# Bell-state tomography in a silicon many-electron artificial molecule

**DOI:** 10.1038/s41467-021-23437-w

**Published:** 2021-05-28

**Authors:** Ross C. C. Leon, Chih Hwan Yang, Jason C. C. Hwang, Julien Camirand Lemyre, Tuomo Tanttu, Wei Huang, Jonathan Y. Huang, Fay E. Hudson, Kohei M. Itoh, Arne Laucht, Michel Pioro-Ladrière, Andre Saraiva, Andrew S. Dzurak

**Affiliations:** 1 School of Electrical Engineering and Telecommunications, The University of New South Wales, Sydney, NSW Australia; 2 Institut Quantique et Département de Physique, Université de Sherbrooke, Sherbrooke, Québec Canada; 3 School of Fundamental Science and Technology, Keio University, Yokohoma, Japan; 4 Quantum Information Science Program, Canadian Institute for Advanced Research, Toronto, ON Canada; 5 Present Address: Quantum Motion Technologies Ltd, London, UK; 6 Present Address: Research and Prototype Foundry, The University of Sydney, Sydney, NSW Australia

**Keywords:** Quantum dots, Qubits

## Abstract

An error-corrected quantum processor will require millions of qubits, accentuating the advantage of nanoscale devices with small footprints, such as silicon quantum dots. However, as for every device with nanoscale dimensions, disorder at the atomic level is detrimental to quantum dot uniformity. Here we investigate two spin qubits confined in a silicon double quantum dot artificial molecule. Each quantum dot has a robust shell structure and, when operated at an occupancy of 5 or 13 electrons, has single spin-$\frac{1}{2}$ valence electron in its *p*- or *d*-orbital, respectively. These higher electron occupancies screen static electric fields arising from atomic-level disorder. The larger multielectron wavefunctions also enable significant overlap between neighbouring qubit electrons, while making space for an interstitial exchange-gate electrode. We implement a universal gate set using the magnetic field gradient of a micromagnet for electrically driven single qubit gates, and a gate-voltage-controlled inter-dot barrier to perform two-qubit gates by pulsed exchange coupling. We use this gate set to demonstrate a Bell state preparation between multielectron qubits with fidelity 90.3%, confirmed by two-qubit state tomography using spin parity measurements.

## Abstract (web summary)

Multielectron quantum dots offer a promising platform for high-performance spin qubits; however, previous demonstrations have been limited to single-qubit operation. Here, the authors report a universal gate set and two-qubit Bell state tomography in a high-occupancy double quantum dot in silicon.

## Introduction

Semiconductor nanodevices, especially those incorporating oxide-insulating layers, suffer from variability due to various atomic-scale defects and morphological imprecision. This disorder degrades spin qubit performance due to the sub-nanometre wave properties of single electrons. The conflict between the benefits of densely packing many quantum dots within a chip and the exposure to disorder demands further research regarding improved systems for encoding solid-state qubits. We exploit here the operation of qubits in silicon metal-oxide-semiconductor (Si-MOS) quantum dots containing several electrons that form closed shells, leaving a single valence electron in the outer shell^1^. The spin of a valence electron in a high-occupancy Si-MOS quantum dot was previously shown to form a high-fidelity single qubit^1^, at least in part due to the improved screening of disorder provided by the raised electron density. However, it was not clear how well two-qubit logic could be performed using such systems, because of the complex molecular states present in a many-electron double quantum dot^2^. We address this here using two multielectron qubits to operate an isolated quantum processing unit^3,4^.

## Results

**Fig. 1:**
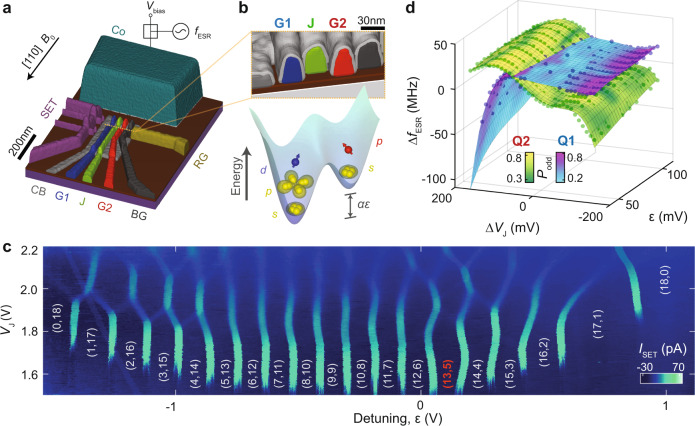
Device overview and electron occupancy measurement. **a** A 3D visualization of the Si-MOS device structure. A quantum dot is formed under gate G1 (blue) and G2 (red), with inter-dot tunnel rates controlled by J (green). Gate RG enables connection to an *n*-doped reservoir to load/unload electrons to/from the quantum dot, with tunnel rates controlled by BG. Gate CB serves as a confinement barrier in lateral direction. The cobalt structure at the top of the image acts as both a micromagnet and electrode for EDSR control (dark green), where a DC voltage bias *V*_bias_ and a microwave signal with frequency *f*_ESR_ is applied. **b** Top: cross-section diagram of **a** along the $[1\bar{1}0]$ crystallographic direction, indicated by the orange dashed line. Bottom: schematic showing the number of electrons in each of the two quantum dots, aligning with the metal gates in the panel above. The height of each electron represents its relative energy and the shell to which it belongs, with inter-dot detuning energy *αε*. Each orbital is labelled correspondingly. Yellow electrons form full shells and are inert, while the extra electron in each dot (blue and red) act as an effective single spin qubit. **c** Charge stability map of the double quantum dot at *B*_0_ = 0 T, showing the charge occupancies (*N*_1_,*N*_2_), produced by plotting the lock-in signal from SET sensor *I*_SET_ as a function of detuning *ε* and *V*_J_. The detuning *ε* = *V*_G1_ − *V*_G2_ is referenced by *ε* = 0 V at the charge readout transition (12,6) ⇔ (13,5). A square wave with peak-to-peak amplitude of 2 mV and frequency 487 Hz is applied to G1 for lock-in excitation. Dynamic compensation is applied to the SET sensor to maintain a high readout sensitivity. **d** Resonance frequency Δ*f*_ESR_ of Q1 and Q2 as a function of *ε* and Δ*V*_J_ = *V*_J_ − 1.58 V when a microwave control pulse of frequency *f*_MW_ = 30.486 GHz + Δ*f*_MW_ is applied. Each data point at any given *ε* and Δ*V*_J_ registers the Δ*f*_MW_ when the adiabatic inversion probability is maximized for each qubit, i.e., when Δ*f*_MW_ = Δ*f*_ESR_. The qubits are initialized as $\left|\downarrow \downarrow \right\rangle$. Colour scale represents the adiabatic inversion probability.

This demonstration is performed with the device structure depicted in Fig. 1a and investigated in previous studies^1,4^. Using the technique adopted from ref. ^4^, where the quantum dots are isolated from the electron reservoir, we load electrons into the two quantum dots formed under gates G1 and G2, and separated by gate J. We monitor inter-dot charge transitions by measuring the transconductance of a nearby single electron transistor (SET). An on-chip cobalt micromagnet is fabricated 120 nm away from the quantum dots. This micromagnet serves two purposes: to create an inhomogeneous magnetic field and an oscillatory electric field, for electrically driven spin resonance (EDSR)^5–7^.

To achieve an isolated mode of operation, the quantum dots are initialized with a desired number of electrons using the reservoir under RG, then the tunnel rate between the quantum dot under G2 and the reservoir is made negligible by lowering the voltage applied to gate BG, such that the double quantum dot becomes isolated^4^. Figure 1c is a charge stability diagram with vertical lines indicating inter-dot charge transition. For the experiment discussed here, we load a total of 18 electrons. It is noteworthy that diagonal lines on the upper half of Fig. 1c (around *V*_J_ = 1.9 V) mark transitions in which the J gate becomes too attractive for electrons and instead of forming a barrier it forms a quantum dot between G1 and G2^4^. At very low voltages, the J gate creates a large barrier between the dots suppressing inter-dot tunnelling. Once the tunnel rate becomes lesser than the lock-in frequency (487 Hz), the transition lines fade, as observed for *V*_J_ < 1.6 V. In contrast to the experiment in ref. ^4^, which was performed using the same device as the present work, but at a lower charge occupancy of (3,3), the inter-dot charge transitions in Fig. 1c exhibits very high uniformity, showing the improved screening of disorder that is possible when many electrons are confined in the quantum dots. As the number of electrons in a quantum dot increases, the dot potential profile becomes more regular and is primarily defined by the gate electrodes structure, with limited impact from random charge disorder.

In a small two-dimensional circular quantum dot, full shells are formed at 4 and 12 electrons^1,8–10^. The fourfold degeneracy of the first shell has its origin in the spin and valley degrees of freedom for silicon conduction band electrons. The next shell is formed by two-dimensional *p*-like states, which means the *p*_*x*_ and *p*_*y*_ states are quasi-degenerate in the approximately circularly symmetric dot. This shell can fit a total of eight electrons. We control the voltage detuning *ε* between gates G1 and G2 voltages such that there are 13 and 5 electrons in Q1 and Q2, respectively, as shown in Fig. 1b, c. This means we have effectively a single valence electron in each quantum dot (*d*-shell and *p*-shell, respectively), whereas the electrons in the inner shells stay inert during spin operations^1^. Evidence supporting the *p*- and *d*-shell structures is demonstrated in the Supplementary Information. In choosing to focus here on the (13,5) charge configuration, we take into consideration the impact that various shell structures have on the qubit performance as identified in refs. ^1,4^. These include the impact of the excited state energies on a number of factors including the following: the creation of relaxation hotspots; the determination of a readout window for the Pauli spin blockades; the EDSR Rabi frequency; and the extent of the wavefunction and how it controls the exchange coupling between neighbouring dots. Early results from ref. ^4^ indicate that choosing the same shell occupancy for both dots does not necessarily imply consistent qubit characteristics, so there is no particular advantage to operating with the same number of electrons in each dot. Therefore, from a proof-of principle perspective, it is beneficial to highlight the versatility of multielectron quantum dots as a qubit platform, by operating a *p*- and a *d*-shell electron in Q1 and Q2, respectively. In an earlier study, we demonstrated the improved performance of these shell configurations compared with *s*- or *f*-shell electrons for single-qubit operation^1^, but a systematic study of the optimal number of electrons for a two-qubit system is out of the scope of our present work.

In general, EDSR control of qubits is heavily influenced by the details of the quantum dot confinement potential^9^. By employing the voltage pulse sequence from ref. ^4^ for initialization, control, and readout, we investigate these parameters performing an adiabatic inversion of the spins with a variable frequency microwave excitation, with an external magnetic field *B*_0_ = 1 T. First, a voltage ramp across the (12,6)–(13,5) transition over a period of 500 μs is applied, which is equivalent to the variation of the detuning *ε*, such that a $\left|\downarrow \downarrow \right\rangle$ spin state is initialized adiabatically. We note that (12,6) provides a good initialization, because it is a spin-0 configuration, as confirmed by magnetospectroscopy (see Supplementary Information). Moreover, a large anticrossing gap between this (12,6) singlet and the $\left|\downarrow \downarrow \right\rangle$ state at (13,5) occupation is created by the difference in quantization axes between dots due to the micromagnet field gradient. We further improve the fidelity of this initialization by simultaneously lowering *V*_J_, to enhance the energy gap between this target state and the (14,4) singlet. Subsequently, a chirped pulse of microwave excitation with variable frequency adiabatically flips one of the spins into an antiparallel configuration, creating either a $\left|\downarrow \uparrow \right\rangle$ or a $\left|\uparrow \downarrow \right\rangle$ state, if the frequency sweep matches the resonance frequency of the qubit^11^. This spin flip is then read out by quickly changing *ε* back to a (12,6) ground state, which will be blockaded by the Pauli principle unless the spin flip to the antiparallel configuration was successful.

Figure 1d shows the nonlinear dependency of the qubit resonance frequencies with electric potentials (Stark shift). Moreover, the efficiency of the adiabatic inversion of the spins depends on the intensity of the effective oscillatory field that drives Rabi oscillations. This is indicated by the colours in Fig. 1d and shows that each qubit has a different optimal gate configuration, such that a sufficiently fast Rabi oscillation frequency is obtained to ensure good control fidelity. This dependence of the Rabi frequency on the gate-voltage configurations was observed previously and associated with the electron position shifting under the micromagnet field^1^. For more information on the method of choosing the optimal operation point, analysis of the Rabi efficiencies, and coherence times of the qubits, refer to Supplementary Information.

**Fig. 2:**
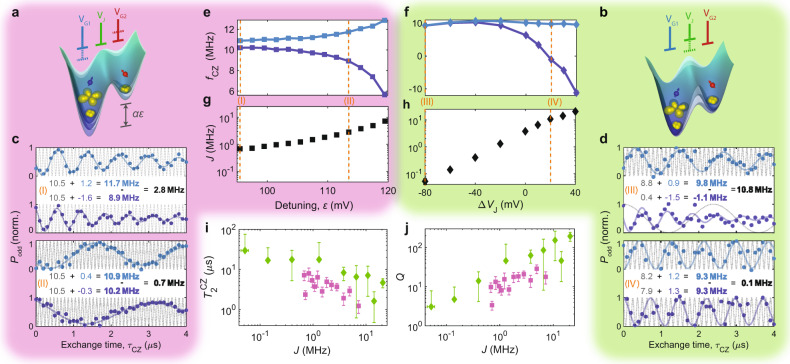
Exchange control. **a**, **b** Schematic showing the two different mechanisms to electrically control exchange coupling between quantum dots, by **a** voltage detuning between G1 and G2 gate, and **b** barrier control with J gate. **c**, **d** Examples of CZ oscillations controlled via **c** detuning or **d** J gate. We apply a pulse sequence X1 − CZ − X1, where X1 is a $\frac{\pi }{2}$ rotation around the *x*-axis of Q1, then measure the probability of an odd spin parity *P*_odd_. Orange Roman numbers in each panel correspond to the applied voltage indicated in **e**–**h**, drawn as orange dashed lines. Blue and red markers correspond to the normalized measured *P*_odd_ with Q2 initialized as $\left|\downarrow \right\rangle$ or $\left|\uparrow \right\rangle$, respectively. The data are fitted using the equation ${P}_{{\rm{odd}}}=\frac{A}{2}(1-\cos (2\pi {f}_{{\rm{osc}}}t){e}^{-t/{T}_{2}^{{\rm{CZ}}}})+b$. The Ramsey frequency *f*_osc_ is displayed as blue or red text on the panel. In order to compensate the strong Stark shift induced by gate pulsing, we adopt different rotating frames, offset by a reference frequency *f*_ref_ between experiments, as presented in grey dashed curves behind each measurement data set. We extract the CZ frequency *f*_CZ_ = *f*_ref_ + *f*_osc_ in a common frame and the difference between ${f}_{{\rm{CZ}},{\rm{Q}}2 = \left|\downarrow \right\rangle }$ and ${f}_{{\rm{CZ}},{\rm{Q}}2 = \left|\uparrow \right\rangle }$, which gives the exchange coupling frequency *J*, shown as black bold text. **e**, **f** The oscillation frequency *f*_CZ_ as a function of **e** detuning or **f** J gate control. Blue and red line corresponds to Q1 =  $\left|\downarrow \right\rangle$ and $\left|\uparrow \right\rangle$, respectively. **g**, **h** Extracted exchange oscillation frequency *J*. **i** Damping time ${T}_{2}^{{\rm{CZ}}}$ of the measured oscillations as a function of exchange coupling *J*, for Q2 = $\left|\uparrow \right\rangle$ and for detuning (purple) and J gate control (green). **j** Quality factor $Q=J\times {T}_{2}^{{\rm{CZ}}}$ as a function of *J*, extracted from **i**. Error bars represents ± 5% fitting error.

The geometry of the MOS device studied here is known to lead to single electron wavefunctions that extend laterally ~10 nm^12^, which is consistent with the large charging energy previously measured in this device when a second electron is added^1^. As the nominal distance from the centre of G1 to the centre of G2 exceeds 60 nm, the inter-dot exchange coupling in the (1,1) charge configuration is predicted to be insufficient for quantum operations—indeed, previous measurements in the same device reveal that exchange is only observed when the J gate is positive enough to form a dot under it^4^. At the *p*- and *d*-shells, nonetheless, the Coulomb repulsion from the core electrons leads to a larger wavefunction for the valence electron. As a result, we are able to measure a sizeable interaction between distant qubits. The ability to control the inter-dot interaction is crucial for high-fidelity two-qubit gate operations^7^. High-fidelity single-qubit gates require low exchange coupling to ensure individual addressibility, whereas two-qubit gates demand strong coupling for fast exchange oscillation with minimal exposure to noise. Previous literature indicates the possibility to control exchange coupling between two multielectron quantum dots^13^. Here we explore two methods for controlling inter-dot interactions—by detuning the quantum dot potentials^14,15^, as shown in Fig. 2a, or by directly controlling the inter-dot barrier potential via an exchange J gate^7,16,17^, as in Fig. 2b.

For each method, the exchange intensity is measured by comparing the precession frequency of one qubit (target) depending on the state of the other qubit (control) with a Ramsey interferometry protocol. Due to the large difference in Larmor frequencies between quantum dots, only the *z* components of the spins couple to each other, while the *x* and *y* components oscillate at different rates for each qubit and their coupling is on average vanishingly small^18,19^. The measured oscillations shown in Fig. 2c, d result from a combination of the exchange coupling and the Stark shift introduced by the gate pulses, measured with regard to a reference frequency *f*_ref_, which can be conveniently chosen to optimize the accuracy of our measurements (see Supplementary Information). The exchange coupling may be obtained by taking the difference between the resulting frequencies for the two states of the control qubit Q2 $\left|\downarrow \right\rangle$ and $\left|\uparrow \right\rangle$.

Figure 2e, f show the extracted oscillation frequencies as controlled by either the detuning *ε* or the exchange-gate voltage *V*_J_. The difference in oscillation frequencies corresponds to the exchange coupling and can be tuned over two orders of magnitude, as seen in the extracted exchange coupling intensities in Fig. 2g, h.We use this conditional control to implement the two-qubit CZ gate. The impact of exchange coupling on qubit coherence is quantified by extracting the decay time of the exchange oscillations ${T}_{2}^{{\rm{CZ}}}$, shown in Fig. 2i as a function of the extracted exchange coupling for both CZ operation methods. We observe an improvement in the driven coherence times when the exchange control is performed by pulsing the J gate to control the inter-dot barrier, as compared to the detuning method. As both methods can reach similar exchange frequencies, this results in an improvement in the quality factor of the exchange oscillations $Q=J\times {T}_{2}^{{\rm{CZ}}}$ as seen in Fig. 2j, similar to previously reported experiments^17,20^. Throughout the rest of this work, we adopt the direct J gate-controlled exchange coupling method for the implementation of CZ logic gates.

**Fig. 3:**
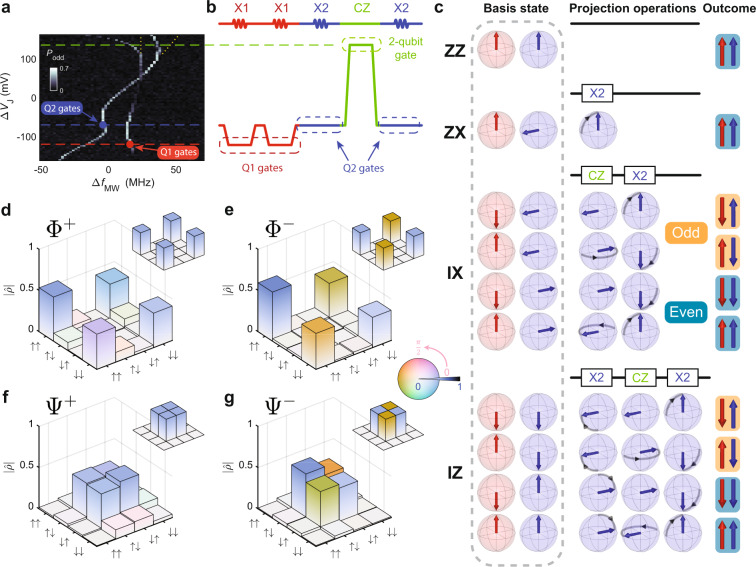
Bell state tomography. **a** Adiabatic inversion probability of both qubits as a function of detuned microwave frequency Δ*f*_MW_, where the carrier frequency is chosen to be the single-qubit operation frequency for Q2 and J gate voltage Δ*V*_J_, with qubits initialized in the $\left|\downarrow \downarrow \right\rangle$ state. Horizontal dashed lines represent J gate voltages applied for various single-qubit and two-qubit gates. Yellow dotted lines are a guide indicating the other resonance frequencies that would be observed at Δ*V*_J_ > 100 mV if the spins were initialized randomly. **b** Schematic of an example microwave and voltage pulse sequence for state tomography. It initializes the qubits as $\left|\uparrow \downarrow \right\rangle$ by performing two $\frac{\pi }{2}$ ×1 pulses (all calibration is performed for $\frac{\pi }{2}$ pulses, such that a high-fidelity *π* pulse is obtained by composing it out of two $\frac{\pi }{2}$ gates, each starting and finishing at a common voltage Δ*V*_J_ = −70 mV, which is shown as a blue dashed line in **a**), then perform IZ projection operation, by converting the parity readout into single-qubit readout via a CNOT gate^4^. Horizontal lines align with Δ*V*_J_ from **a**. **c** Example qubit states and operations required to obtain projections along the indicated axes. The first, two columns of Bloch spheres represent the eigenstates of Q1 (red) and Q2 (blue) before state tomography, whereas the rest illustrates the logic gate operations required for state tomography, before parity readout. For IX and IZ, all possible initial eigenstates are displayed, with parity results shown on the last column. **d**–**g** Quantum-state tomography of Bell states **d** ${{{\Phi }}}^{+}=\frac{\left|\uparrow \uparrow \right\rangle +\left|\downarrow \downarrow \right\rangle }{\sqrt{2}}$, **e** ${{{\Phi }}}^{-}=\frac{\left|\uparrow \uparrow \right\rangle -\left|\downarrow \downarrow \right\rangle }{\sqrt{2}}$, **f** ${{{\Psi }}}^{+}=\frac{\left|\uparrow \downarrow \right\rangle +\left|\downarrow \uparrow \right\rangle }{\sqrt{2}}$, **g** ${{{\Psi }}}^{-}=\frac{\left|\uparrow \downarrow \right\rangle -\left|\downarrow \uparrow \right\rangle }{\sqrt{2}}$. The height of the bars represents the absolute value of density matrix elements, whereas complex phase information is encoded in the colour map. Inset: bar graph of the ideal density matrix of the corresponding Bell state. The measured fidelities of each Bell state are (87.1 ± 2.8)%, (90.3 ± 3.0)%, (90.3 ± 2.4)%, and (90.2 ± 2.9)%, from **d** to **g**, respectively.

As shown in Fig. 1d, both qubits possess a strongly nonlinear Stark shift and large variation in the efficiency of the EDSR drive. Single-qubit control fidelity in excess of 99% was only achieved when the gate-voltage configuration was tuned differently for each qubit, as indicated in the example gate sequence shown in Fig. 3a. This leads to a major limitation—single-qubit gates must be performed sequentially, whereas the other qubit is left idling^21^, unable to be protected by refocusing techniques such as dynamical decoupling^14,22^ or pulse shaping^23^. Together with the two-qubit CZ gate, these gates span the two-qubit Clifford space (see Fig. 3b for illustration).

The strong Stark shift between operating points leads to a phase accumulation with regard to a reference frequency, which must be accounted for in gate implementations (see Supplementary Information). To minimize the gate error introduced by resonance frequency shifts (due to electrical 1/*f* noise and ^29^Si nuclear spin flips), a number of feedback protocols are implemented. The following input parameters are monitored periodically and adjusted if necessary: SET bias voltage, readout voltage level, ESR frequencies of both qubits, phase accumulations at five different gate voltages for the logic gates, and exchange coupling. This results in a total of ten feedback calibrations in each experiment. Further information on phase and exchange coupling feedback is provided in the Supplementary Information.

We gauge the quality of our gate set implementation by preparing Bell states and evaluating them through two-qubit state tomography^24,25^. For a double quantum dot isolated from the reservoir, parity readout is used for the measurements^4,26^, which implies that a readout step will contain the collective information of both qubits or, more precisely, the ZZ projection of the two qubits. To read out other projections, single- and two-qubit gate operations can be performed before readout. Figure 3c displays some key examples of such tomography protocols. The gate sequence illustrated in Fig. 3b represents the example of an IZ measurement, which maps the spin state of the second qubit into the parities of the two-spin arrangement, regardless of the initial state of the first spin. To completely reconstruct the 4 × 4 density matrix of a two-qubit system, 15 linearly independent tomography projections are required^27^ (the complete list is presented in the Supplementary Information). The results for each Bell state are shown in Fig. 3d–g. The state preparation fidelities range from 87.5% to 90.3%, which compares favourably with state-of-the-art two spin qubit systems^3,7,28^.

## Discussion

Our study highlights various advantages of multielectron qubits, which lead to efficient EDSR-based single-qubit gates and extended reach of the exchange coupling between neighbouring qubits. The protocol for logic gates developed here leads to promising fidelities for Bell state preparation, but its use in longer computations would be impacted by the inability to refocus the spin that is not being manipulated. This problem can be solved by designing a more efficient EDSR strategy without the need to optimize the gate configuration, or by using an antenna to produce microwave magnetic field-based electron spin resonance^29^. The ability of additional core electrons to screen charge disorder at the Si/SiO_2_ interface^2,30^, as demonstrated here, indicates that multielectron qubits offer a promising pathway for near term demonstrations of quantum processing in silicon.

## Supplementary information

Supplementary Information

## Data Availability

The data that support the findings of this study are available from the authors on reasonable request; see author contributions for specific data sets.

## Code availability

The code that support the findings of this study are available from the authors on reasonable request; see author contributions for specific code sets.
