# Supplementary material for: Bell-state tomography in a silicon many-electron artificial molecule
Source: Nat Commun. 2021 May 28;12:3228. doi: 10.1038/s41467-021-23437-w (PMC8163798; doi:10.1038/s41467-021-23437-w)
Supplement: Supplementary file 1 — Supplementary Information [file 41467_2021_23437_MOESM1_ESM.pdf]

Supplementary Information: Bell-state tomography in a silicon many-electron artificial molecule

Leon et al.

CONTENTS

Supplementary Note 1: Magnetospectroscopy of an isolated double quantum dot 2

Supplementary Note 2: Adiabatic inversion and qubit operation points 2

Supplementary Note 3: Exchange oscillation, coherence and Q factors of interacting spins 4

Supplementary Note 4: Measurement feedback 4

Supplementary Note 5: Exchange coupling feedback 5

Supplementary Note 6: Two qubit tomography with parity readout 6

Supplementary Note 7: Fidelity estimation 8

References 9

SUPPLEMENTARY NOTE 1: MAGNETOSPECTROSCOPY OF AN ISOLATED DOUBLE QUANTUM DOT

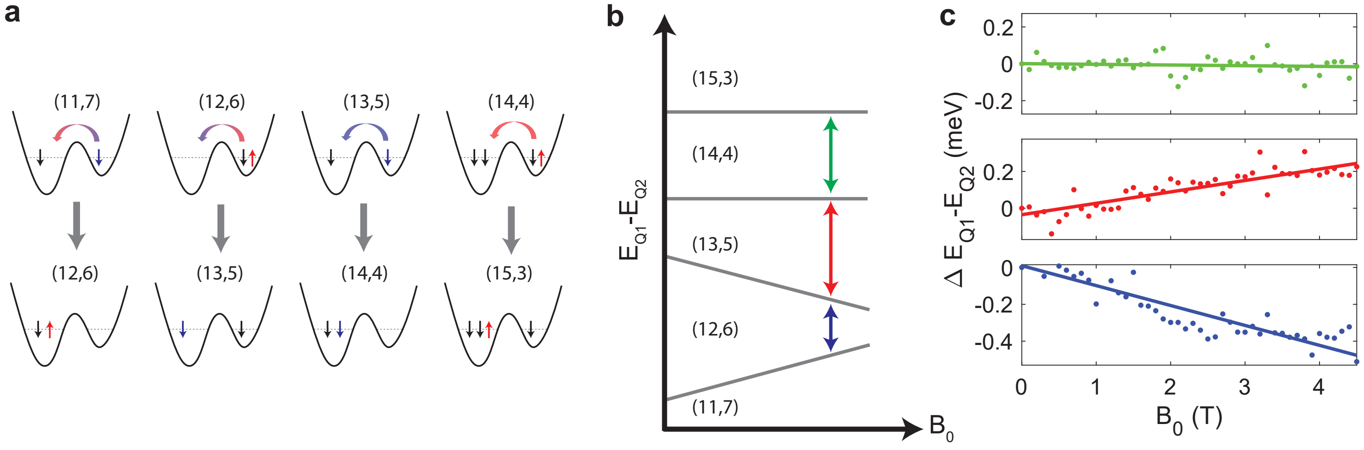

Supplementary Figure 1 | Magnetospectroscopy of an isolated double quantum dot. **a**, Estimated spin state of the active valence electrons before (top row of schematics) and after (bottom row of schematics) an inter-dot charge transition at corresponding electron number (m,n), where m and n represent the total number of electrons in Q1 and Q2 respectively. Coloured arrows represents the electron which participates in charge transition, with blue and red indicate spin down and up, respectively. **b**, Illustration of energy difference between Q1 and Q2 as a function of applied magnetic field B_0, as corresponding electron numbers in each dot. **c**, Extracted experimental magnetospectroscopy data, with each colour corresponding to the energy difference in charge transition shown in **(b)**.

From the single dot shell structure [1], one can try to predict which double dot occupations will lead to a single spin $\frac{1}{2}$ qubit in each dot. But in order to confirm that the spin structure of the double dot can be extrapolated from single dot results, we obtain the spin ordering of the dots performing magnetospectroscopy. Traditionally, magnetospectroscopy is performed studying the shifts of chemical potentials of each dot as a function of the externally applied magnetic field. This assumes that the quantum dot is in diffusive equilibrium with a reservoir (same chemical potential). Such reservoir is assumed to be spinless, such that its chemical potential does not shift with magnetic field and the absolute shift in dot chemical potential with magnetic field can be assessed. In our system, the two dots are in equilibrium with each other, but all transitions conserve the total number of electrons in the double dot system (isolated double dot) – there is no reference reservoir, as shown in Supplementary Figure 1a. Therefore, only relative Zeeman shifts are observed.

The hypothetical field dependencies, assuming that the shell structure from Ref. 1 holds, are shown in the energy diagram in Supplementary Figure 1b. The measured magnetospectroscopy results in Supplementary Figure 1c confirm our assumption. In particular, the (13,5) charge configuration consists indeed of single spin-$\frac{1}{2}$ states in both dots, each atop an inert closed shell of spin 0.

Note that the lever arm we extracted from the slope in Supplementary Figure 1c is the sum of lever arm from Q1 and Q2, approximately $\alpha_{Q1} + \alpha_{Q2} = 0.53\,\text{eV/V}$. Differences in lever arm $\alpha_{Q1} - \alpha_{Q2}$ cannot be obtained from this method.

SUPPLEMENTARY NOTE 2: ADIABATIC INVERSION AND QUBIT OPERATION POINTS

In order to achieve single qubit EDSR control fidelities exceeding 99 %, compliant with the demands for quantum error correction in the surface code architecture, we must adjust the inter-dot detuning and J gate voltage such that we achieve the most efficient Rabi drive for both Q1 and Q2.

We perform an adiabatic spin inversion experiment by sweeping the microwave frequency applied to the EDSR gate electrode (in our case the Co magnet) at fixed power, such that when each of the qubit resonance frequencies f_{ESR} is found, that spin is flipped with an efficiency given by the comparison between the sweeping speed and the Rabi frequency (limited by the spin relaxation time) [2]. This is observed as an increase in the probability of measuring an odd parity readout after preparing the even initial state $|\downarrow\downarrow\rangle$, with an example shown in Figure 3a. This permits

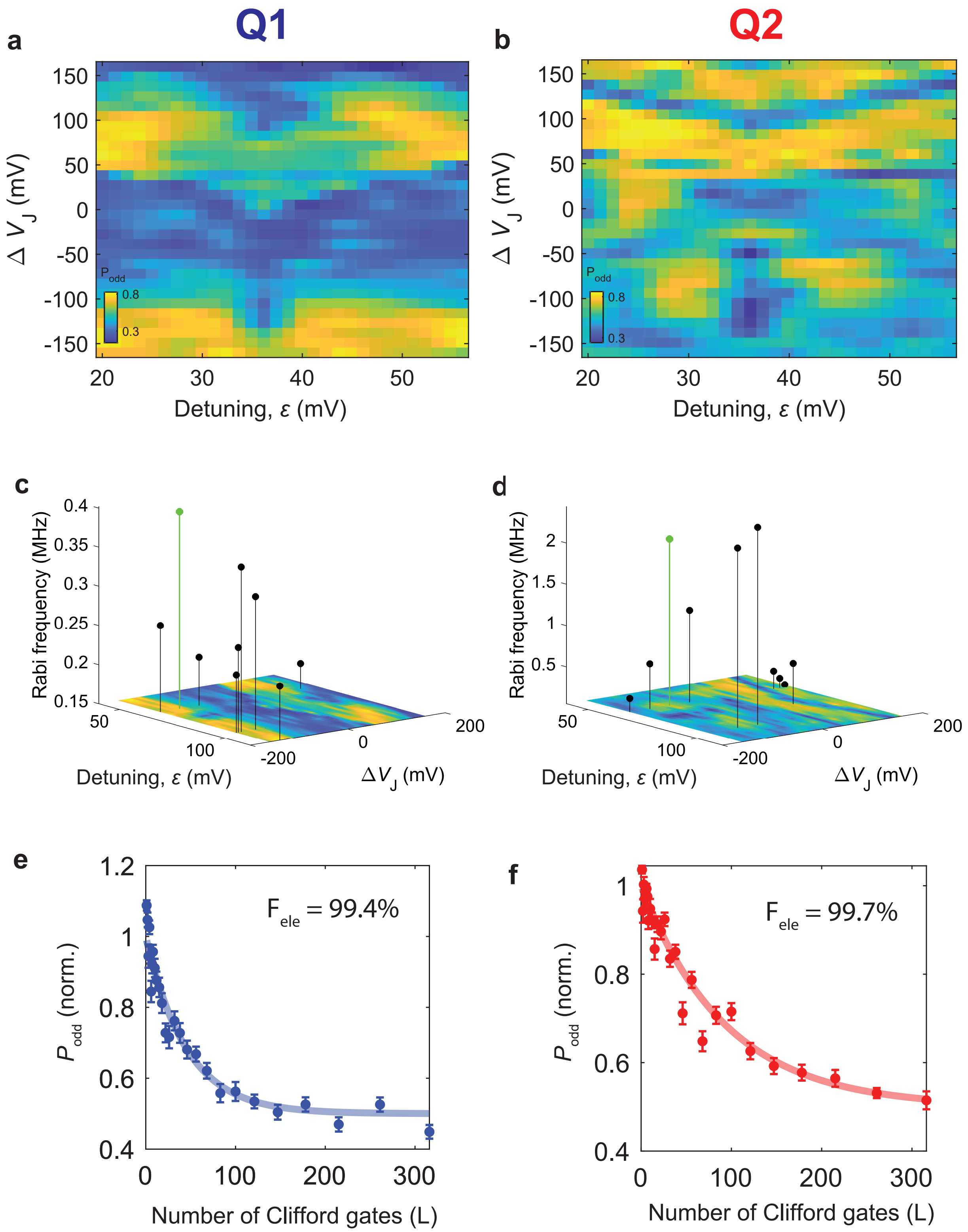

Supplementary Figure 2 | Single qubit operation voltage. a,b, Adiabatic inversion probability of **(a)** Q1 or **(a)** Q2 as a function of detuning and J gate voltage, with interpolation. **c,d**, Rabi frequency f_{Rabi} for selected detuning and J gate voltage combinations, with the 2D plot of panels **(a)** and **(b)** copied at bottom of x-y plane. **e,f**, Single qubit randomised benchmarking for **(e)** Q1 or **(f)** Q2 at voltages at the green marker in panels **(c)** and **(d)**, respectively. Error bars represent the standard error of the mean.

us to determine the resonance frequencies, as well as the region of high qubit fidelity, as a function of detuning and J gate voltage.

The colour scale in Figure 1d shows the extracted adiabatic inversion probability of each qubit at various detuning and J gate voltages. We interpolated these probabilities and plotted them again in Supplementary Figure 2a and b. At first glance, we notice that $P_{\rm odd}$ is symmetric along the axis of detuning $\varepsilon = 37\,\mathrm{mV}$, implying that detuning the dots in either direction has the same effect on dot shape and spin behaviour.

The strategy to quickly calibrate the ideal operation points is to choose a few potential operation points on the 2D map where $P_{\rm odd}$ shows a high adiabatic inversion probability, and measure the Rabi oscillation frequency at a fixed microwave power. We then choose the highest Rabi frequency point that meets some constrains. Firstly, for individual addressability by frequency modulation, the ESR frequency $f_{\rm ESR}$ of both qubits should be at least 10 MHz apart, which means $\Delta V_{\rm J} < 20\,\mathrm{mV}$ or $> 100\,\mathrm{mV}$ in Figure 3a. Also, we would like to minimise the exchange coupling during single qubit operation, which is achieved for $\Delta V_{\rm J} < -20\,\mathrm{mV}$, setting $J < 1\,\mathrm{MHz}$ as observed from Figure 2h. As a result, we are generally limited to the bottom half of the 2D map in Supplementary Figure 2a and b. Ideally, we would like to choose an optimal operation point such that we can perform single qubit operation on both Q1 and Q2 (see main text for detail). However, there is no observable voltage range from Supplementary Figure 2a and b where both qubits gives high $P_{\rm odd}$ under the constrains mentioned above.

A few detuning and J gate voltage combinations with $P_{\rm odd} > 0.42$ are chosen for each qubit, and Rabi frequencies are extracted in Supplementary Figure 2c and d. The green markers from the plots are the operation points chosen for single qubit randomised benchmarking, with results presented in Supplementary Figure 2e and f. Qubits Q1 and Q2 have control fidelities $F_{\rm Q1} = (99.40 \pm 0.17)\,\%$ and $F_{\rm Q2} = (99.70 \pm 0.10)\,\%$, respectively. Note that the operation point chosen for Q2 is not the one with the absolute maximum Rabi frequency, as we also would like to minimise gate voltage fluctuation when ramping between Q1 and Q2 logic gate operations. We observe a significant influence of ramping range on the final outcome of the Bell state preparation, but a thorough evaluation of this source of error is not warranted, since this relates to instrument limitations.

Coherence times T_2^* for Q1 and Q2 at the chosen operation points are $(13.7 \pm 2.0)\,\mu\mathrm{s}$ and $(8.4 \pm 3.3)\,\mu\mathrm{s}$, respectively, while $T_2^{\rm Hahn}$ are $(50.0 \pm 15.2)\,\mu\mathrm{s}$ and $(94.6 \pm 18.7)\,\mu\mathrm{s}$, respectively.

SUPPLEMENTARY NOTE 3: EXCHANGE OSCILLATION, COHERENCE AND Q FACTORS OF INTERACTING SPINS

The oscillations observed from Ramsey-like experiments in the main text Figure 2c, d are due to difference in precession frequency of the qubits in the period between $\frac{\pi}{2}$-pulses. The difference in frequencies arises from both Stark shift, which is in the order of 10 MHz in our experiments, and exchange coupling J, between 100 kHz and 10 MHz. As a result, the total Ramsey frequency will be dominated by Stark shift, making the J-coupling effect difficult to observe without a high resolution scan of precession time. Therefore, we adjust the phase of the second $\frac{\pi}{2}$-pulse to match a rotating frame of reference which is not the same as the qubit Q1 precession frequency $f_{\rm Q1}$, but instead it is offset by a value $f_{\rm ref}$ chosen to reduce the impact of the Stark shift to the oscillation observed in experiment. This reference frequency is adjusted *ad hoc* between different experiments in order to facilitate the extraction of the exchange coupling effect.

In the left panel of Figure 2, where the quantum dots are detuned, $f_{\rm ref}$ is set to 10.5 MHz throughout the experiment. However, for direct J gate controlled CZ, the oscillation frequency varies across a range of 20 MHz, as shown in Figure 2f. In order to capture the oscillation data efficiently, we assign various $f_{\rm ref}$ for each $\Delta V_{\rm J}$ targeting a shift of approximately -1 MHz from the CZ frequency $f_{\rm CZ}$ (which could differ depending on whether the control spin is up or down).

In a qubit rotating frame, positive and negative phase accumulation will result in the same Ramsey oscillation if only a single measurement projection is taken. To determine the sign of ESR frequency shift, we repeat every Ramsey experiment with additional phase shift on the second $\frac{\pi}{2}$ pulse, in order to extract X,−X,Y,−Y projections of the qubit. Note that all four measurements are taken in a interleaved fashion to minimise the impact of quasi-static noise.

SUPPLEMENTARY NOTE 4: MEASUREMENT FEEDBACK

Low frequency noise is a major limitation for high fidelity operation of qubits in MOS devices [3]. An efficient approach to mitigate high amplitude noise that occurs in a sub-Hz scale is to recalibrate the most critical qubit control parameters periodically.

There are 10 parameters that require feedback throughout the experiments due to the intricate way by which the qubit operations are defined with different gate configurations targeting the optimisation of each qubit. These parameters are the SET Coulomb peak alignment, the readout level set by the dot gate, both qubit ESR frequencies, a total of five relative phases acquired when pulsing between operating points, and the exchange coupling controlled by the J gate. The SET feedback is used to maintain its high sensitivity during charge transition, while read level feedback is to ensure the readout is done within a Pauli spin blockade region for parity readout. SET and readout level feedbacks are performed with first order corrections, with a predefined target SET current. SET top gate voltage V_{ST} and read level voltage (controlled via V_{G1}) are updated based upon the difference between measured current and target current.

We adopt the ESR frequency tracking protocol from Ref. 3 in order to follow the resonance frequency jumps due to quasi-static noise such as hyperfine coupling with residual ^{29}Si nuclear spin in the silicon wafer, as well as low frequency electrical noise. We perform checks of each of the two resonance frequencies shown in Figure 3a independently every 10 measurement data points. If the spin rotation is unsuccessful at the assumed resonance frequency, we recalibrate the frequency with a series of Ramsey experiments.

In Figure 3a, the ESR frequency shift Δf_{ESR} is taken as 0 MHz at the microwave driving frequency that matches the resonance frequency of Q2 at voltage $\Delta V_{\mathrm{J}} = -70$ mV, which is the operating point for Q2. At all the other operation points where Δf_{ESR} is non-zero, a phase will accumulate due to variations in precession frequency. Since our Clifford set requires 3 operation voltages, each with two phases for Q1 and Q2 to track, excluding the reference frequency $f_{\mathrm{ESR}} = f_{\mathrm{Q2}}$, that results in 5 phase accumulations to recalibrate.

Although phase accumulation can be calculated by the extracted ESR frequency (Δf_{ESR}) and gate time t_{g}, i.e. $\phi = \Delta f_{\mathrm{ESR}} \times t_{\mathrm{g}}$, such method assumes an instantaneous step from one gate voltage to another, which in reality is limited by the 80 MHz bandwidth of the measurement cable, meaning during the ramp both qubits spend a non-negligible amount of time in an intermediate voltage state, accumulating phases that are non-trivial to calculate, especially when the Stark shift is highly non-linear as seen in Figure 3a. Moreover, it is unclear whether the low frequency noise will affect the overall shape of the gate dependency of the resonant frequencies.

In quantum computing, all operations can be performed by a sequence of gates taken from a primitive gate set. The processing unit is fully calibrated if all the primitive gates are calibrated individually. Supplementary Table 1 shows the pulse sequences required to extract each of the 5 phases accumulated, each associate with certain qubit and primitive gates.

Level	ΔV_{J} (mV)	Q1	target gate	Q2	target gate	Primitive gate
1	-120	$\mathrm{X1}^2$	I1	X2–I1–X2	I1	X1,Y1
2	-70	X1–I2–X1	I2	N/A	N/A	X2,Y2
3	130	X1–CZ–X1	CZ	X2–CZ–X2	CZ	CZ

Supplementary Table 1 | Pulse sequences for qubit phase calibration. Pulse sequences used to extract phase accumulation while idling. ΔV_{J} (mV) is referenced from Figure 3a. Element at column Qn row ΔV_{J} corresponds to pulse sequence required to extract phase accumulated in qubit n when inter-dot barrier gate voltage is at ΔV_{J}. Rn represents a $\frac{\pi}{2}$ rotation around R-axis on qubit n, with $R \in \{\mathrm{X}, \mathrm{Y}\}$, while I$n$ means identity gate with ΔV_{J} equals to the voltage where single qubit operation is performed for qubit n.

Phase calibration is performed every ten measurements, after the ESR frequencies are updated. In each calibration, the corresponding pulse sequence from Supplementary Table 1 is applied with various phases ϕ for the last $\frac{\pi}{2}$ pulse with respect to the other pulses. The results are then fitted with a function $P_{\mathrm{odd}} = A\cos(2\pi(\phi - \phi')) + b$, where A and b are fitting constants related to the oscillation visibility and dark counts, while ϕ' is the phase accumulated from the target gate. Since this protocol may rely on multiple primitive gates in a sequence, the phase associated with each gate in Supplementary Table 1 has to be calibrated following a certain order , to ensure the phase extracted corresponds to one particular primitive gate only. These phases ϕ' will be used for compensation of unwanted accumulated phases as we apply the corresponding Clifford gates in the experiment.

SUPPLEMENTARY NOTE 5: EXCHANGE COUPLING FEEDBACK

The exchange coupling J may fluctuate between experiments due to low frequency electrical noise, which can be compensated by monitoring and recalibrating the CZ gate operation with a feedback protocol. The sampling rate of the arbitrary waveform generator (AWG) and microwave IQ modulation used here, 8 ns and 10 ns respectively, limit our gate operation times to the least common multiple of these two, $\tau_{\mathrm{CZ}} = 40$ ns, or any multiples of that. This means

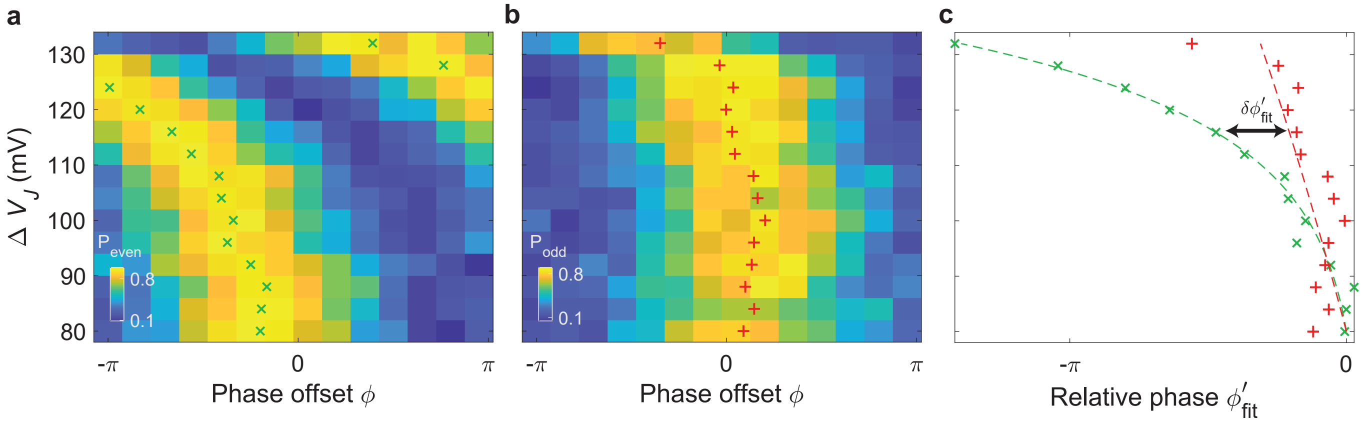

Supplementary Figure 3 | Phase accumulation from CZ operation. **a,b**, Parity readout probability as a function of exchange gate voltage ΔV_J and phase offset ϕ, for duration of $\tau_{CZ} = 160\,$ns, with gates **(a)** X1−CZ−X1(ϕ) or **(b)** $X2^2$−X1−CZ−X1(ϕ) applied. ϕ represent the phase offset of the second X1 pulse with respect to the first within the same sequence. Marker in each row indicate the fitted phase ϕ' from a $P_{even/odd} = A\cos(2\pi(\phi - \phi')) + b$, where A,b and ϕ' are constants. Note that since the control qubit is initilised into opposite spin state and parity readout is used, opposite parity is extracted for the two cases in order to obtain the same single spin information from the target qubit. **c**, Fitted phase ϕ' from **(a)** (green '×') and **(b)** (red '+'), which are fitted with equation $\phi'_{fit} = A\exp(-b\Delta V_J) + c$, where A, b and c are constants. Both graphs are offset to zero phase at $\Delta V_J = 80\,$mV.

that updating the CZ exchange time τ_{CZ} is not accurate enough for high fidelity operation. Instead, we update the inter-dot barrier gate voltage V_J, which compensates the change in J while leaving τ_{CZ} unchanged.

The initial calibration method is as follows: two CZ identical sequences are performed, each one with an opposite control qubit state (spin down or up). We vary the readout projection angles ϕ and fit the parity readout probability to a sinusoidal wave similar to the case of the phase feedback, which we use to extract the phase offset ϕ'_{fit}. The difference in phase accumulated in the control spin down and up cases are due to the composition of an exchange coupling from the CZ operation and from the extra $X2^2$ gate necessary for the control spin up calibration step. The latter can be compensated by re-scaling ϕ'_{fit} to 0 at low exchange coupling regime.

This experiment is repeated with various exchange gate voltages ΔV_J, as shown in Supplementary Figure 3a and b, while the resulting phases, are plotted on Supplementary Figure 3c, along with an exponential fit. The difference between the two lines in Supplementary Figure 3c are the phase contributed from exchange coupling J, which can be calculated from $J = \frac{\delta\phi'_{fit}}{\tau_{CZ}}$.

Upon choosing the desired value of J with the associated ΔV_J, which should correspond to a $\delta\phi'_{fit} = \pi$ phase difference between the two initial states, a feedback protocol can be implemented to recalibrate J periodically. The feedback protocol is similar to the initial calibration mentioned above, but optimised for speed by focusing on a smaller range of ΔV_J, and the exponential fit used in Supplementary Figure 3c is replaced with a linear fit. With that, the value of ΔV_J is updated using the fit in order to maintain the same exchange coupling strength J.

This exchange coupling feedback is performed after ten measurements, immediately after the phase calibration step. Note that the pulse sequence used in Supplementary Figure 3a is identical to the one in Supplementary Table 1. Therefore, the X1−CZ−X1 sequence is omitted from the phase calibration stage, but extracted from the subsequent exchange coupling feedback stage.

Supplementary Figure 4 is an example of a Bell state tomography experiment, with all ten feedback loops active, and the variation of the respective parameters over 40 minutes of laboratory time. The parameters that are calibrated only every ten measurements have larger gaps between data points.

SUPPLEMENTARY NOTE 6: TWO QUBIT TOMOGRAPHY WITH PARITY READOUT

A two qubit density matrix is a 4×4 matrix spanning a $4^2 - 1 = 15$ dimensional space and requires 15 linearly independent projection measurements. Ref. 4 gives a detailed explanation on how to perform two-qubit state tomography using parity readout. Supplementary Table 2 lists the gate operation sequences adopted here for each of the 15 projection measurements, using a combination of primitive gates described in the main text.

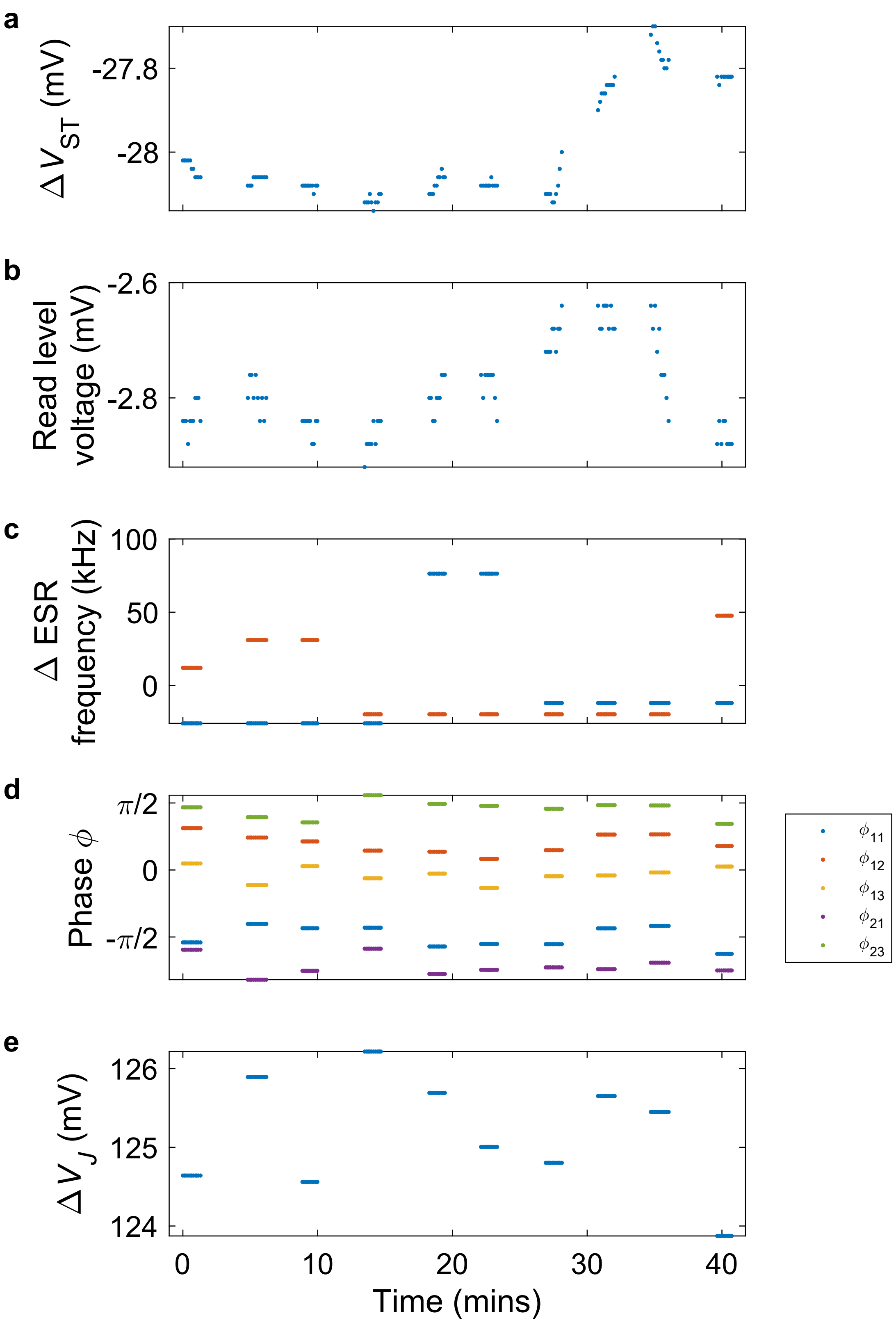

Supplementary Figure 4 | Parameters tracking over measurement time. Various parameters are recorded while Bell state tomography of Figure 3g is running. **a**, ST gate voltage ΔV_{ST} from SET feedback. **b**, G1 gate voltage ΔV_{G1} during readout, from parity readout feedback. **c**, Change in resonance frequency Δ_{ESR} for Q1 (blue) and Q2 (red). **d**, Phase accumulation ϕ_{MN} from the target gate in Supplementary Table 1, where M and N represents the level and qubit in the table, respectively. **e**, J gate voltage ΔV_{J} required to maintain a phase difference of $\delta\phi'_{\mathrm{fit}} = \pi$ at $\tau_{\mathrm{CZ}} = 160\,\mathrm{ns}$ during CZ operation.

Projection	Operations
ZZ	I
YZ	X1
XZ	Y1
ZY	X2
ZX	Y2
YY	X1−X2
YX	X1−Y2
XY	Y1−X2
XX	Y1−Y2
YI	CZ−X1
XI	CZ−Y1
IY	CZ−X2
IX	CZ−Y2
ZI	X1−CZ−X1
IZ	X2−CZ−X2

Supplementary Table 2 | Gate operations for parity readout. List of operations required for a complete state tomography via parity readout, with each row representing the projection axis of interest for a two-qubit system, and the sequence of gate operations required prior to readout.

SUPPLEMENTARY NOTE 7: FIDELITY ESTIMATION

In order to accurately estimate the fidelity of the control steps in preparing a Bell state, some post-processing techniques are applied to the outcome of the measured odd parity probability P_{odd} corresponding to the 15 projections from Supplementary Table 2.

Firstly, we factor in the errors associated with state initialisation and measurement (SPAM error), by renormalising the parity readout probability of the two qubits for ZZ readout.

Next, we reconstruct the density matrix from the measurement data. Let E_υ be the measurement outcome projector, ρ be density matrix, p_υ be the measurement probability, where $\upsilon = 1...30$ (notice that measurements of the projector P_{MN}, where $M, N \in \{\mathrm{I}, \mathrm{X}, \mathrm{Y}, \mathrm{Z}\}$, produce not only probability p_{MN}, but also $p_{-MN} = 1 - p_{MN}$, so that 15 projections yield 30 probabilities). We define a matrix A as

$$A = \begin{pmatrix} \vec{E}_1^\dagger \\ \vec{E}_2^\dagger \\ \vdots \\ \vec{E}_{30}^\dagger, \end{pmatrix} \tag{1}$$

where $\vec{E}_\upsilon^\dagger$ stands for the vectorised form of the projection E_υ.

Similarly, all elements of ρ can also be vectorised. This yields the relation:

$$\begin{aligned} A\vec{\rho} = \begin{pmatrix} \vec{E}_1^\dagger\vec{\rho} \\ \vec{E}_2^\dagger\vec{\rho} \\ \vdots \\ \vec{E}_{30}^\dagger\vec{\rho} \end{pmatrix} &= \begin{pmatrix} \mathrm{tr}\{E_1^\dagger\rho\} \\ \mathrm{tr}\{E_2^\dagger\rho\} \\ \vdots \\ \mathrm{tr}\{E_{30}^\dagger\rho\} \end{pmatrix} = \begin{pmatrix} P(E_1|\rho) \\ P(E_2|\rho) \\ \vdots \\ P(E_{30}|\rho) \end{pmatrix} \\ \approx \begin{pmatrix} p_1 \\ p_2 \\ \vdots \\ p_{30} \end{pmatrix} &= \vec{p} \end{aligned} \tag{2}$$

With matrix A constructed from our choice of measurement projection, and $\vec{p}$ from measurement data. We then perform a (pseudo) linear inversion to estimate the density matrix $\hat{\rho}$.

Since the matrix computed numerically by linear inversion can be an unphysical state for a qubit (leading to a measured matrix $\vec{p}$ that does not have the properties of a density matrix), a maximum likelihood technique is used to numerically estimate the density matrix [5] under several constrains. A legitimate qubit density matrix must be

non-negative definite, have a trace of one and be Hermitian. These conditions are met if we write the density matrix as [5]:

$$\hat{\rho} = \frac{T^\dagger T}{\mathrm{tr}\{T^\dagger T\}} \tag{3}$$

where

$$T = \begin{pmatrix} t_1 & 0 & 0 & 0 \\ t_5 + it_6 & t_2 & 0 & 0 \\ t_{11} + it_{12} & t_7 + it_8 & t_3 & 0 \\ t_{15} + it_{16} & t_{13} + it_{14} & t_9 + it_{10} & t_4 \end{pmatrix} \tag{4}$$

and $t_1..t_{16}$ are real numbers. To find these values, we apply a maximum likelihood estimation, with the cost function

$$L(t_1, t_2, ...t_{16}) = \sum_v \frac{(\langle\psi_v|\hat{\rho}(t_1, t_2, ...t_{16})|\psi_v\rangle - n_v)^2}{2\,\langle\psi_v|\hat{\rho}(t_1, t_2, ...t_{16})|\psi_v\rangle} \tag{5}$$

where ψ_v is the vectorised measurement matrix with $v = 1...30$ and n_v are the measurement probabilities. We start our search inputing the density matrix resulting from the pseudo-linear inversion described before and proceed to numerically optimise L as a function of $t_1, t_2, ...t_{16}$. The resulting elements will give our final density matrix.

The fidelity of a Bell state is calculated then from the definition $F(\rho, \hat{\rho}) = (\mathrm{tr}\{\sqrt{\sqrt{\rho}\hat{\rho}\sqrt{\rho}}\})^2$, where ρ and $\hat{\rho}$ are the ideal and measured density matrices, respectively.

REFERENCES

[1] Leon, R. C. C. *et al.* Coherent spin control of s-, p-, d- and f-electrons in a silicon quantum dot. *Nature Communications* **11**, 797 (2020).

[2] Laucht, A. *et al.* High-fidelity adiabatic inversion of a ^{31}P electron spin qubit in natural silicon. *Applied Physics Letters* **104**, 092115 (2014).

[3] Huang, W. *et al.* Fidelity benchmarks for two-qubit gates in silicon. *Nature* **569**, 532–536 (2019).

[4] Seedhouse, A. E. *et al.* Pauli blockade in silicon quantum dots with spin-orbit control. *PRX Quantum* **2**, 010303 (2021).

[5] Altepeter, J. B., James, D. F. & Kwiat, P. G. 4 qubit quantum state tomography. In Paris, M. & Řeháček, J. (eds.) *Quantum State Estimation*, Lecture Notes in Physics, 113–145 (Springer, Berlin, Heidelberg, 2004).
